# Role of MicroRNAs in Obesity-Induced Metabolic Disorder and Immune Response

**DOI:** 10.1155/2018/2835761

**Published:** 2018-02-01

**Authors:** Hong Zhong, Minjuan Ma, Tingming Liang, Li Guo

**Affiliations:** ^1^Jiangsu Key Laboratory for Molecular and Medical Biotechnology, College of Life Sciences, Nanjing Normal University, Nanjing, Jiangsu 210023, China; ^2^Department of Bioinformatics, School of Geographic and Biologic Information, Nanjing University of Posts and Telecommunications, Nanjing, Jiangsu 210023, China

## Abstract

In all living organisms, metabolic homeostasis and the immune system are the most fundamental requirements for survival. Recently, obesity has become a global public health issue, which is the cardinal risk factor for metabolic disorder. Many diseases emanating from obesity-induced metabolic dysfunction are responsible for the activated immune system, including innate and adaptive responses. Of note, inflammation is the manifest accountant signal. Deeply studied microRNAs (miRNAs) have participated in many pathways involved in metabolism and immune responses to protect cells from multiple harmful stimulants, and they play an important role in determining the progress through targeting different inflammatory pathways. Thus, immune response and metabolic regulation are highly integrated with miRNAs. Collectively, miRNAs are the new targets for therapy in immune dysfunction.

## 1. Introduction

Obesity is the result of imbalanced energy intake and expenditure, which is defined as abnormal or excessive ectopic fat accumulation in peripheral tissues that may impair health. It is estimated that by 2030, the overweight adults (body mass index (BMI) > 25 kg/m^2^) are projected to be 1.35 billion, and 573 million of these are considered clinically obese (BMI > 30 kg/m^2^) in the world [1]. Obesity plays an important role in the dysfunction of the liver, cardiac, pulmonary, endocrine, and reproductive systems, resulting in serious metabolic disorders, such as diabetes, fatty liver disease, atherosclerosis, and some cancers. This imposes a spectacular burden on personal health, society, and economy. Treatments of the escalating obesity and metabolic disorder have been a long journey which requires efforts from each level of society. Further, medical therapy and surgery are also powerful measures to shape tackling and curbing programs.

Since the beginning of life, metabolic response and immune system are highly interwoven for tissue and organismal health. It was reported that immune cells, such as macrophages and mast cells, infiltrated adipose tissue in obese animal models [2], suggesting an immunological nature of metabolic disease. This observation can clarify another study which showed that some diabetic patients treated with aspirin exhibited rapid improvement in glucose homeostasis [3]. On the other hand, dysimmunity is paramount for metabolic disorder. Fox et al. reported that patients with meningitis exhibited a transient diabetic syndrome [4]. Another study also found that treatment with lipopolysaccharide in dogs caused resistance to insulin by abrogating the ability of insulin to induce glucose uptake in the muscle [5]. Besides, it was recognized that acute infection in human patients was associated with decreased binding of insulin to the insulin receptor in isolated blood cells [6]. Hence, delicate regulation of these pathways is vital for cell homeostasis.

miRNAs are small noncoding, endogenous, single-stranded RNAs usually consisting of 18–25 nucleotides that regulate gene expression through repression or degradation of targeted mRNAs at the posttranscriptional level [7]. It is estimated that about 30–50% of protein-coding genes are regulated by miRNAs [8]. Disrupted expression of miRNAs participating in cell process is related to many diseases, such as obesity-induced hyperlipidemia, nonalcoholic fatty liver disease (NAFLD), type 2 diabetes mellitus (T2DM), and atherosclerosis, through regulation of multiple genes [9]. In the immune system, the feedback networks serve to regulate protein expression at a steady state and conditions of environmental stress that are necessary for fate commitment [10]. Therefore, miRNAs implicated in immune system development and function have a potential role in the therapeutics for immune-related diseases.

In this review, we focus on obesity-induced metabolic disorder with the goal to illustrate the links with immune response and the role of miRNAs and therefore to develop effective therapeutic strategies.

## 2. miRNAs and Obesity-Induced Metabolic Disorder

Obesity is the primary target for prevention and treatment as elevated serum concentration of lipid in obese subjects may lead to severe disturbances (lipotoxicity) and inevitable metabolic disorder. Adipose tissue is the main organ for lipid storage; however, excess calories will change endocrine functions of adipocytes and the ectopic fat accumulation in peripheral tissues, such as liver, skeletal muscle, pancreatic *β*-cells, and kidney [11], will lead to lipotoxic stress and low-grade inflammation, accompanied by metabolic disorder.

In the last few years, there has been a growing interest in the role of miRNAs in the development of obesity-induced metabolic disorder. These miRNAs play important roles in physiologic and pathophysiological conditions which participate in cell differentiation, proliferation, apoptosis, hematopoiesis, limb morphogenesis, and important metabolic pathways, such as insulin secretion, triglyceride and cholesterol biosynthesis, and oxidative stress [12, 13]. Among these, it is shown that miR-103, miR-107, and miR-143 accelerate fat cell development [14]. miR-935, miR-4772, miR-223, and miR-376b are reporters of diet-induced obesity [15]. And mice lacking miR-378 are resistant to obesity and exhibit enhanced mitochondrial fatty acid metabolism and elevated oxidative capacity in insulin target tissues [16]. miR-221, miR-28, and miR-486 are associated with BMI, percentage fat mass, waist, and regional fat distribution [17]. In addition, miR-126, miR-15a, miR-29b, miR-223, and miR-28-3p are related to T2DM, and miR-155, miR-302a, and miR-712 are related to atherosclerosis [18].

## 3. Inflammation and Immune Response in Obesity-Induced Metabolic Disorder

The living organisms activate the immune system composed of cell lineages residing in lymphoid organs or vary tissues and transit through the peripheral blood against infectious pathogens. Inflammation is a self-protective response with the goal to clear antigens and return the system back to a normal baseline, which recruits leukocytes to fat, but lacks many of the cardinal signs of classic inflammation, such as dolor, rubor, calor, and tumor. Immune activation is recurrent in superimposed metabolic disorder on obesity with tonic low-grade inflammation. Innate and adaptive immune responses are different kinds of immunity interacting with additional cells to form dynamic cellular communities in tissues. Innate immunity is an intrinsic, cell-autonomous response representing the first barrier of fast-acting defense against pathogens, while adaptive immune response stimulates antigen-specific receptor molecules expressed by T and B lymphocytes [19]. Excess lipid in obese individuals is the main cause of metabolic disorder. Likewise, it may also influence the ability of the immune system. Thus, it is vital to evaluate the role of immune response and inflammation in the obesity-induced metabolic disorder.

All metabolic tissues contain resident populations of immune cells, and all cells with normal metabolism perform cell-type-specific biological functions involved in immune responses against ambient environment [20], which gave birth to the concepts of “immunometabolism” [21] and “metainflammation” [22]. Immunometabolism is proposed to depict metabolism connected to immunity and the metabolic impact on immune cell function, while metainflammation is a discipline of chronic low-grade inflammatory response to obesity.

**Figure 1:**
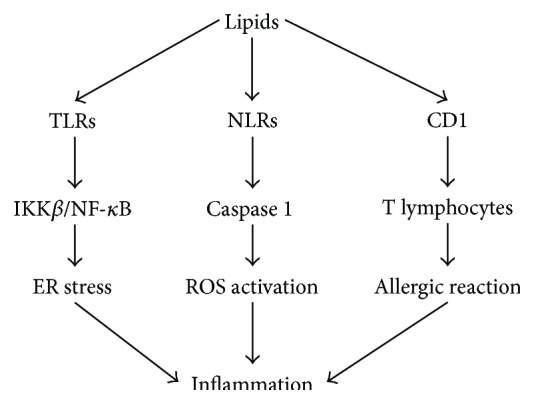
Inflammation pathways induced by lipid. Lipid can be recognized by multiple molecules resident in cellular membrane, such as TLRs, NLRs, and CD1, to activate different signal pathways and ultimately induce inflammation. TLRs are responsible for IKK*β*/NF-*κ*B and ER stress augment, while NLRs activate Caspase1 expression and induce ROS production. Besides, lipid captured by CD1 can be presented to T lymphocytes directly. All these signals participate in translating lipids to inflammatory response.

There are multiple signaling pathways participating in promoting obesity-derived diseases and involved in the progress of inflammation. Lipid can act directly on cells of the innate system to promote the development of Th2-type responses associated with allergy or through CD1 to capture and present lipid antigen restricted to T lymphocytes, which can promote allergic reactions [23, 24]. Macrophages are important for lipid sensing and induction of the inflammatory programming from an anomalous activation of the innate immune system. In the presence of a continuous nutritional surplus, foreign pathogen molecules such as lipid or saturated fatty acids are sensed by lipid transporter, pattern recognition receptors (PRRs, such as Toll-like receptors (TLRs), and Nod-like receptors (NLRs)) or other cytokine receptors to initiate a defense response. Intracellular lipids are recognized to ligate several immune receptors by TLRs and subsequently induce inflammatory activity and inflammatory gene transcription, resulting in the production and secretion of cytokines such as tumor necrosis factor (TNF) and interleukin 6 (IL-6) [25], which are overexpressed in the adipose tissue of obese mice providing the first clear link between obesity and induced metabolic disorder [26]. I*κ*B kinase-*β* as the downstream target and activation of IKK*β*/NF-*κ*B is crucial in inflammation in the obese state. Besides, in insulin-responsive tissues, JNK is activated by fatty acids, insulin, hyperglycemia, and inflammatory cytokines [27]. Another downstream pathway is endoplasmic reticulum (ER) stress, which activates unfolded protein response and governs multiple metabolic responses [28]. In addition, lipid recognized by NLRs activated Caspase1 and ultimately resulted in ROS activation and the release of IL-1*β* and IL-18 [29]. Thus, the lipid accumulation tissues which are populated by macrophages and other immune cells give rise to chronic activation of inflammatory pathways in the setting of obesity (Figure 1). Further work is needed to show the precise cell signal for deep understanding of the response against pathogen infections.

### 3.1. Hyperlipidemia and Atherosclerosis

Hyperlipidemia, a chronic disorder with high levels of triglyceride (TG, hypertriglyceridemia), total cholesterol (TC, hypercholesterolemia), and low-density lipoprotein cholesterol (LDLC) and a decreased level of high-density lipoprotein cholesterol (HDLC), is a manifest consequence of obesity. Lipid droplets are absorbed by intestine cells and transported to tissues for storage and expenditure. Thus, regulation of lipid absorption, generation, and expenditure is crucial in determining circulating lipid levels. To understand the prevalence of hyperlipidemia in China, Li et al. determined TG, TC, HDLC, and LDLC levels in fasting serum for 97,409 subjects who were selected by multistage stratified cluster random sampling from 162 surveillance points of 31 provinces in 2010. After the complex weighting, data showed that prevalence of hypertriglyceridemia, hypercholesterolemia, high blood LDLC, and low blood HDLC in Chinese adults was 11.3%, 3.3%, 2.1%, and 44.8%, respectively [30].

Atherosclerosis is a result of fatty streak lesions initiated by macrophages forming foam cells trapped beneath the endothelial cell lining in the artery [31]. It is enhanced after continued recruitment of immune cells and subsequent proliferation and migration of smooth muscle cells to larger fibrofatty plaques, followed with significant narrowing of the arterial lumen, leading to chronic syndromes, such as cardiovascular disease [32]. The major clinical manifestations of atherosclerosis include ischemic heart disease, ischemic stroke, and peripheral arterial disease. It is the leading cause of death worldwide which is declared by the World Health Organization to highlight its prevalence threat to public health.

Atherosclerotic lesions recruit inflamed endothelial cells in postcapillary venues, such as intracellular adhesion molecule-1, E-selectin, and vascular cell adhesion molecule-1. Macrophage scavenger receptor type A expressed by immune cells recognizes and facilitates the phagocytosis of specific surface molecules of pathogens. Besides, CD36 and TLRs are also receptors regulated by macrophages and endothelial cells contributing to inflammation [33, 34], which can provide a link between systemic inflammation and local infection in driving plaque growth or engendering atherosclerotic plaque instability. The role of inflammatory cytokines and mediators influence the development of atherosclerotic lesions [35]. In addition, interferon-*γ* and IL-18 are two Th1 cytokines involved in proatherogenic reaction. Recent studies show that IL-18 receptor is expressed in multiple immune cells within human atherosclerotic plaques, while intraperitoneal injection of recombinant IL-18 increased atherosclerotic-lesion size twofold in *ApoE^−/−^* mice [36, 37].

### 3.2. NAFLD

NAFLD is a pathologic syndrome ranging from simple steatosis through steatohepatitis to fibrosis and cirrhosis which are characterized by excess fat accumulation in hepatocytes that is associated with an enlargement of the liver (hepatomegaly) accompanied by inflammation, leading to loss of metabolic competency as reduced mitochondrial *β*-oxidation capacity and induced endoplasmic reticulum stress, oxidative stress, and hepatocyte apoptosis. It is the major risk factor of chronic liver disease in the developed countries as the prevalence of steatosis in patients with obesity is about 75% [38]. In a US community, the incidence of NAFLD diagnosis increased 5-fold from 1997 to 2014 [39]; therefore, it was expected that within the next decade, NAFLD-associated hepatic disorder could be the most common. In general, nearly 10–20% of NAFLD patients will progress to nonalcoholic steatohepatitis (NASH) and 8–25% of NASH patients may develop liver cirrhosis. Up to 2.8% of NASH cases may further develop into end-stage liver disease or hepatocellular carcinoma [40].

NAFLD is characterized of hepatic lipid accumulation accompanied with inflammation. Acute immune response and coordinated network of multiple cell types are essential for maintaining metabolic homeostasis. In lipid accumulation tissues, aggregation macrophages predominantly assume a classical proinflammatory activation state (M1) through Th1 responses while reducing an alternative macrophage activation state (M2) generated by Th2 cytokines which promotes fibrotic responses [41, 42], resulting in the suppressed recruitment of eosinophils and attenuation of classical NF-*κ*B-dependent activation pathways [43, 44], leading to low-grade inflammation. Many of the signaling pathways such as TLR, JNK, and ER stress were elevated in steatotic liver inducing inflammation and metabolic dysfunction. In addition, M1/Th1 cytokines are increased mediated by immune cells recruited to the liver.

### 3.3. T2DM

Glucose homeostasis is controlled by multiple organ system, including brain, pancreas, and peripheral tissues (such as liver, adipose tissue, and skeletal muscle). In the fasted state, release of glucose from liver is a key for euglycemia. Circulatory glucose originates from hydrolysis of glycogen (the polysaccharide storage form of glucose) in the liver as well as from gluconeogenesis (de novo production of glucose from non-glucose-derived carbon precursors). As a compensatory response to postprandial hyperglycemia, plasma insulin concentration rises to maintain normal glucose homeostasis by inducing glucose uptake in the skeletal muscle and liver while simultaneously inhibiting hepatic glucose production [45]. However, nutritional excess enhances the secretion of insulin but blunts the response of organs to insulin and ultimately results in the clinical manifestation of T2DM, the most studied multifactorial metabolic disorder associated with obesity. The global prevalence of T2DM is rapidly increasing, and epidemiologists predict that the number of patients will double in the second half of the twentieth century by 2030 in China [46]. The number of people with diabetes mellitus is projected to rise to 439 million globally, which represents 7.7% of the total adult population of the world's adults [47].

T2DM is relevant to synergistic action by multiple organs, such as pancreatic islets, liver, adipose tissue, and skeletal muscle, which coordinated to determine circulatory glucose level and insulin action. Pancreatic islets are the critical cells for insulin secretion. When the lipid is overwhelmed, macrophages are recruited and produce proinflammatory cytokines to induce inflammation, which result in blunted *β*-cell function, reduced insulin secretion, and cell apoptosis, leading to decreased islet mass. Excess lipid in liver, adipose tissue, and skeletal muscle has causal relationship with insulin resistance. The increased adipose tissue mass is related to an estimated excess of 20–30 million macrophages that accumulate with each kilogram of excess fat in humans [48]. The inflammatory cytokines are increased in obesity coupled with myocytes' capacity in response to inflammatory and metabolism [49, 50]. Infiltrating macrophages accumulated in muscle induce M1 activation [51].

Taken together, at the molecular and cellular levels, excess nutrients such as lipid can induce secretion of cytokines and trigger inflammatory responses in obesity-induced disorder.

## 4. Role of miRNAs in Metabolic Disorder and Immunity

There are many miRNAs enriched in immune response dysfunction to affect immunity [52]. miR-125 has a vital role in maintaining normal inflammatory cytokine output, which targets several mRNAs that are important in development and apoptosis, thereby altering immune cell biology in complex ways. Overexpression of miR-125a decreases cell apoptosis and increases total number of bone marrow cells [53]. Beyond this, many others have been linked to the modulation of immune cell development. A recent study shows that ectopic expression of miR-142 has been found to increase production of T lymphocytes *in vitro* [54]. Besides, miR-221 and miR-222 are downregulated during erythropoiesis, thus relieving repression of their target, which encodes the stem cell factor receptor c-Kit [55].

In addition to function on gene expression participating in immune response, miRNAs also influence metabolism. For example, miR-100, miR-130, and miR-155 which is positive in macrophage infiltration are inhibited with adipocyte differentiation [18]. miR-155 is ubiquitously expressed, not only in many haemopoietic cell types but also in human reproductive tissues, fibroblasts, epithelial tissues, and central nervous system [56]. The miR-155 is encoded by a gene originally isolated near a common retroviral integration site-induced lymphomas [57]. It is found that this miRNA is upregulated in atherosclerosis which is coordinated with lipid and inflammation [58]. Also, its expression is downregulated in mature immune cells and increased in adaptive macrophages after exposure to inflammatory cytokines [59, 60]. The importance of proper regulation of miR-155 expression is exemplified by its much higher expression in response to infection [61–63].

miR-33 is the typical miRNA abundant in lipoprotein particles which is crucial in lipid metabolism [64]. Targets of miR-33 include key enzymes of fatty acid uptake and metabolism such as CPT-1, AMPK, and *β*-hydroxyacyl-CoA dehydrogenase [65]. Moreover, overexpression of miR-33 significantly inhibits cellular fatty acid oxidation and enhances mitochondrial oxidative capacity and ATP production [66, 67]. Further, numerous studies have regarded miR-33 as the therapy target of obesity and induced metabolic disorder [68]. At the same time, a recent study shows that miR-33 regulates the innate immune response via ATP-binding cassette transporter [69]. Consistent with this, *Abca1^−/−^* and *Abcg1^−/−^* macrophages have increased TLR proinflammatory responses, which indicate that miR-33 augment TLR signaling in macrophages via a raft cholesterol-dependent mechanism. Another study shows that miR-33 controls adaptive fibrotic response in the remodeling heart by preserving lipid raft cholesterol [70].

## 5. Conclusions and Future Therapeutic Directions

miRNAs are now widely regarded as playing a critical role in regulating homeostasis of obesity-induced metabolic disorder and immune response by fine tuning the expression of a network of genes through posttranscriptional regulation. Specific miRNA expression profiles can be utilized as biomarkers for diagnosis, prognostic purposes, and clinical development in various diseases [71]. However, studies in demonstrating the therapy role of miRNAs in metabolic disorder and dysimmunity are lagging.

**Figure 2:**
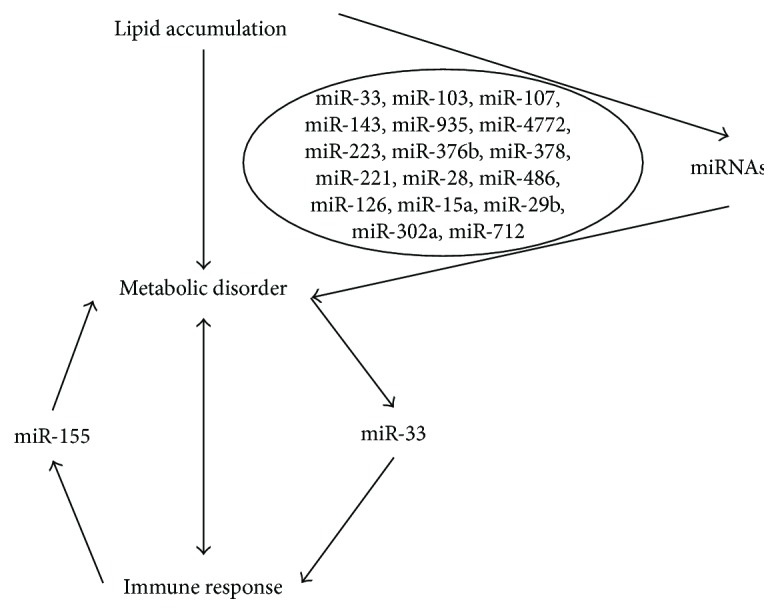
Role of miRNAs in obesity-induced metabolic disorder and immune response. Many miRNAs are regulated by lipid accumulation and play a mediatory effect in coupling obesity and metabolic disorder. Moreover, they also mediate the induction of immune response. Here, we illustrated two typical miRNAs to show the critical role of miRNAs in the interactions of metabolic disorder and immune response.

In this review, we analyze the important role of miRNAs in obesity-induced metabolic disorder and immune response. We listed many diseases induced by obesity, such as NAFLD, T2DM, hyperlipidemia, and atherosclerosis, which have affinity with miRNAs. These miRNAs participate in many pathways and regulate metabolism progression, including insulin secretion, triglyceride and cholesterol biosynthesis, and oxidative stress. Moreover, metabolic disorder accounts for dysimmunity as ectopic and excess lipid accumulation in cell can be detected by multiple signals, such as TLRs, NLRs, and CD1, to initiate inflammatory response. And dysimmunity is accompanied by metabolic disorder as patients with meningitis exhibited an instant diabetic syndrome. Further, miRNAs play a crucial role in coupling metabolism and immunity. As shown before, miRNAs regulated by immune response can regulate the development of obesity-induced metabolic disorder. On the contrary, immune response regulated by metabolism is mediated by miRNAs (Figure 2).

Thus, it is expected that a better understanding of miRNAs in obesity-induced disorder and immune response will lead to the discovery of the potential therapy role of miRNAs in metabolic and immune-related disorder. And further work needs to accelerate the clinical use of miRNAs.

## Abbreviations

BMI: Body mass index
ER: Endoplasmic reticulum
HDLC: High-density lipoprotein cholesterol
IL: Interleukin
LDLC: Low-density lipoprotein cholesterol
miRNAs: MicroRNAs
NAFLD: Nonalcoholic fatty liver disease
NASH: Nonalcoholic steatohepatitis
NLRs: Nod-like receptors
PRRs: Pattern recognition receptors
TC: Total cholesterol
TG: Triglyceride
TLRs: Toll-like receptors
TNF: Tumor necrosis factor
T2DM: Type 2 diabetes mellitus.

## References

[1] Kelly T., Yang W., Chen C. S., Reynolds K., He J. (2008). Global burden of obesity in 2005 and projections to 2030. *International Journal of Obesity*.

[2] Hausberger F. X. (1966). Pathological changes in adipose tissue of obese mice. *The Anatomical Record*.

[3] Turkoglu S., Abaci A. (2007). Use of aspirin in diabetic patients. *Anadolu Kardiyoloji Dergisi*.

[4] Fox M. J., Kuzma J. F., Washam W. T. (1947). Transitory diabetic syndrome associated with meningococcic meningitis. *Archives of Internal Medicine*.

[5] Raymond R. M., Harkema J. M., Emerson T. E. (1981). In vivo skeletal muscle insulin resistance during E coli endotoxin shock in the dog. *Circulatory Shock*.

[6] Drobny E. C., Abramson E. C., Baumann G. (1984). Insulin receptors in acute infection: a study of factors conferring insulin resistance. *The Journal of Clinical Endocrinology & Metabolism*.

[7] Bartel D. P. (2004). MicroRNAs: genomics, biogenesis, mechanism, and function. *Cell*.

[8] Filipowicz W., Bhattacharyya S. N., Sonenberg N. (2008). Mechanisms of post-transcriptional regulation by microRNAs: are the answers in sight?. *Nature Reviews Genetics*.

[9] Vienberg S., Geiger J., Madsen S., Dalgaard L. T. (2017). MicroRNAs in metabolism. *Acta Physiologica*.

[10] Na Y. J., Kim J. H. (2013). Understanding cooperativity of microRNAs via microRNA association networks. *BMC Genomics*.

[11] Unger R. H. (2003). Minireview: weapons of lean body mass destruction: the role of ectopic lipids in the metabolic syndrome. *Endocrinology*.

[12] Aranda J. F., Madrigal-Matute J., Rotllan N., Fernandez-Hernando C. (2013). MicroRNA modulation of lipid metabolism and oxidative stress in cardiometabolic diseases. *Free Radical Biology & Medicine*.

[13] Hulsmans M., De Keyzer D., Holvoet P. (2011). MicroRNAs regulating oxidative stress and inflammation in relation to obesity and atherosclerosis. *The FASEB Journal*.

[14] Xie H., Lim B., Lodish H. F. (2009). MicroRNAs induced during adipogenesis that accelerate fat cell development are downregulated in obesity. *Diabetes*.

[15] Milagro F. I., Miranda J., Portillo M. P., Fernandez-Quintela A., Campion J., Martinez J. A. (2013). High-throughput sequencing of microRNAs in peripheral blood mononuclear cells: identification of potential weight loss biomarkers. *PLoS One*.

[16] Carrer M., Liu N., Grueter C. E. (2012). Control of mitochondrial metabolism and systemic energy homeostasis by microRNAs 378 and 378. *Proceedings of the National Academy of Sciences of the United States of America*.

[17] Prats-Puig A., Ortega F. J., Mercader J. M. (2013). Changes in circulating microRNAs are associated with childhood obesity. *The Journal of Clinical Endocrinology & Metabolism*.

[18] Marques-Rocha J. L., Samblas M., Milagro F. I., Bressan J., Martinez J. A., Marti A. (2015). Noncoding RNAs, cytokines, and inflammation-related diseases. *The FASEB Journal*.

[19] Stubbington M. J. T., Rozenblatt-Rosen O., Regev A., Teichmann S. A. (2017). Single-cell transcriptomics to explore the immune system in health and disease. *Science*.

[20] Lumeng C. N., Deyoung S. M., Bodzin J. L., Saltiel A. R. (2007). Increased inflammatory properties of adipose tissue macrophages recruited during diet-induced obesity. *Diabetes*.

[21] Hotamisligil G. S. (2017). Foundations of immunometabolism and implications for metabolic health and disease. *Immunity*.

[22] Hotamisligil G. S. (2006). Inflammation and metabolic disorders. *Nature*.

[23] Moody D. B., Cotton R. N. (2017). Four pathways of CD1 antigen presentation to T cells. *Current Opinion in Immunology*.

[24] Del Moral M. G., Martinez-Naves E. (2017). The role of lipids in development of allergic responses. *Immune Network*.

[25] Gregor M. F., Hotamisligil G. S. (2011). Inflammatory mechanisms in obesity. *Annual Review of Immunology*.

[26] Hotamisligil G. S., Shargill N. S., Spiegelman B. M. (1993). Adipose expression of tumor necrosis factor-alpha: direct role in obesity-linked insulin resistance. *Science*.

[27] Solinas G., Karin M. (2010). JNK1 and IKK*β*: molecular links between obesity and metabolic dysfunction. *The FASEB Journal*.

[28] Hotamisligil G. S. (2010). Endoplasmic reticulum stress and the inflammatory basis of metabolic disease. *Cell*.

[29] Ives A., Nomura J., Martinon F. (2015). Xanthine oxidoreductase regulates macrophage IL1*β* secretion upon NLRP3 inflammasome activation. *Nature Communications*.

[30] Li J. H., Wang L. M., Li Y. C. (2012). Epidemiologic characteristics of dyslipidemia in Chinese adults 2010. *Zhonghua Yu Fang Yi Xue Za Zhi*.

[31] Libby P. (2001). Current concepts of the pathogenesis of the acute coronary syndromes. *Circulation*.

[32] Greaves D. R., Channon K. M. (2002). Inflammation and immune responses in atherosclerosis. *Trends in Immunology*.

[33] Febbraio M., Podrez E. A., Smith J. D. (2000). Targeted disruption of the class B scavenger receptor CD36 protects against atherosclerotic lesion development in mice. *The Journal of Clinical Investigation*.

[34] Edfeldt K., Swedenborg J., Hansson G. K., Yan Z. Q. (2002). Expression of toll-like receptors in human atherosclerotic lesions: a possible pathway for plaque activation. *Circulation*.

[35] Glass C. K., Witztum J. L. (2001). Atherosclerosis. The road ahead. *Cell*.

[36] Gerdes N., Sukhova G. K., Libby P., Reynolds R. S., Young J. L., Schonbeck U. (2002). Expression of interleukin (IL)-18 and functional IL-18 receptor on human vascular endothelial cells, smooth muscle cells, and macrophages: implications for atherogenesis. *Journal of Experimental Medicine*.

[37] Whitman S. C., Ravisankar P., Daugherty A. (2002). Interleukin-18 enhances atherosclerosis in apolipoprotein E^−/−^ mice through release of interferon-*γ*. *Circulation Research*.

[38] Anderson N., Borlak J. (2008). Molecular mechanisms and therapeutic targets in steatosis and steatohepatitis. *Pharmacological Reviews*.

[39] Allen A. M., Terry T. M., Larson J. J., Coward A., Somers V. K., Kamath P. S. (2017). Nonalcoholic fatty liver disease incidence and impact on metabolic burden and death: a 20 year-community study. *Hepatology*.

[40] Lee J., Kim Y., Friso S., Choi S. W. (2017). Epigenetics in non-alcoholic fatty liver disease. *Molecular Aspects of Medicine*.

[41] Shaul M. E., Bennett G., Strissel K. J., Greenberg A. S., Obin M. S. (2010). Dynamic, M2-like remodeling phenotypes of CD11c+ adipose tissue macrophages during high-fat diet--induced obesity in mice. *Diabetes*.

[42] Aron-Wisnewsky J., Tordjman J., Poitou C. (2009). Human adipose tissue macrophages: m1 and m2 cell surface markers in subcutaneous and omental depots and after weight loss. *The Journal of Clinical Endocrinology and Metabolism*.

[43] Lumeng C. N., Bodzin J. L., Saltiel A. R. (2007). Obesity induces a phenotypic switch in adipose tissue macrophage polarization. *The Journal of Clinical Investigation*.

[44] Wu D., Molofsky A. B., Liang H. E. (2011). Eosinophils sustain adipose alternatively activated macrophages associated with glucose homeostasis. *Science*.

[45] Shulman G. I., Rothman D. L., Jue T., Stein P., DeFronzo R. A., Shulman R. G. (1990). Quantitation of muscle glycogen synthesis in normal subjects and subjects with non-insulin-dependent diabetes by ^13^C nuclear magnetic resonance spectroscopy. *The New England Journal of Medicine*.

[46] Ginter E., Simko V. (2012). Type 2 diabetes mellitus, pandemic in 21st century. *Advances in Experimental Medicine and Biology*.

[47] Chen L., Magliano D. J., Zimmet P. Z. (2011). The worldwide epidemiology of type 2 diabetes mellitus--present and future perspectives. *Nature Reviews Endocrinology*.

[48] O'Rourke R. W., Metcalf M. D., White A. E. (2009). Depot-specific differences in inflammatory mediators and a role for NK cells and IFN-*γ* in inflammation in human adipose tissue. *International Journal of Obesity*.

[49] Saghizadeh M., Ong J. M., Garvey W. T., Henry R. R., Kern P. A. (1996). The expression of TNF alpha by human muscle. Relationship to insulin resistance. *The Journal of Clinical Investigation*.

[50] Frisard M. I., McMillan R. P., Marchand J. (2010). Toll-like receptor 4 modulates skeletal muscle substrate metabolism. *American Journal of Physiology Endocrinology and Metabolism*.

[51] Patsouris D., Li P. P., Thapar D., Chapman J., Olefsky J. M., Neels J. G. (2008). Ablation of CD11c-positive cells normalizes insulin sensitivity in obese insulin resistant animals. *Cell Metabolism*.

[52] Mehta A., Baltimore D. (2016). MicroRNAs as regulatory elements in immune system logic. *Nature Reviews Immunology*.

[53] O'Connell R. M., Chaudhuri A. A., Rao D. S., Gibson W. S., Balazs A. B., Baltimore D. (2010). MicroRNAs enriched in hematopoietic stem cells differentially regulate long-term hematopoietic output. *Proceedings of the National Academy of Sciences of the United States of America*.

[54] Chen C. Z., Li L., Lodish H. F., Bartel D. P. (2004). MicroRNAs modulate hematopoietic lineage differentiation. *Science*.

[55] Felli N., Fontana L., Pelosi E. (2005). MicroRNAs 221 and 222 inhibit normal erythropoiesis and erythroleukemic cell growth via kit receptor down-modulation. *Proceedings of the National Academy of Sciences of the United States of America*.

[56] Teng G., Papavasiliou F. N. (2009). Shhh! Silencing by microRNA-155. *Philosophical Transactions of the Royal Society Series B, Biological Sciences*.

[57] Tam W., Ben-Yehuda D., Hayward W. S. (1997). bic, a novel gene activated by proviral insertions in avian leukosis virus-induced lymphomas, is likely to function through its noncoding RNA. *Molecular and Cellular Biology*.

[58] Ma X., Ma C., Zheng X. (2013). MicroRNA-155 in the pathogenesis of atherosclerosis: a conflicting role?. *Heart, Lung and Circulation*.

[59] Taganov K. D., Boldin M. P., Chang K. J., Baltimore D. (2006). NF-*κ*B-dependent induction of microRNA miR-146, an inhibitor targeted to signaling proteins of innate immune responses. *Proceedings of the National Academy of Sciences of the United States of America*.

[60] Thai T. H., Calado D. P., Casola S. (2007). Regulation of the germinal center response by microRNA-155. *Science*.

[61] Eis P. S., Tam W., Sun L. (2005). Accumulation of miR-155 and BIC RNA in human B cell lymphomas. *Proceedings of the National Academy of Sciences of the United States of America*.

[62] Fulci V., Chiaretti S., Goldoni M. (2007). Quantitative technologies establish a novel microRNA profile of chronic lymphocytic leukemia. *Blood*.

[63] Costinean S., Zanesi N., Pekarsky Y. (2006). Pre-B cell proliferation and lymphoblastic leukemia/high-grade lymphoma in E*μ*-miR155 transgenic mice. *Proceedings of the National Academy of Sciences of the United States of America*.

[64] Najafi-Shoushtari S. H., Kristo F., Li Y. (2010). MicroRNA-33 and the SREBP host genes cooperate to control cholesterol homeostasis. *Science*.

[65] Goedeke L., Vales-Lara F. M., Fenstermaker M. (2013). A regulatory role for microRNA 33 in controlling lipid metabolism gene expression. *Molecular and Cellular Biology*.

[66] Rayner K. J., Esau C. C., Hussain F. N. (2011). Inhibition of miR-33a/b in non-human primates raises plasma HDL and lowers VLDL triglycerides. *Nature*.

[67] Karunakaran D., Richards L., Geoffrion M. (2015). Therapeutic inhibition of miR-33 promotes fatty acid oxidation but does not ameliorate metabolic dysfunction in diet-induced obesity. *Arteriosclerosis, Thrombosis, and Vascular Biology*.

[68] Rayner K. J., Sheedy F. J., Esau C. C. (2011). Antagonism of miR-33 in mice promotes reverse cholesterol transport and regression of atherosclerosis. *The Journal of Clinical Investigation*.

[69] Lai L., Azzam K. M., Lin W. C. (2016). MicroRNA-33 regulates the innate immune response via ATP binding cassette transporter-mediated remodeling of membrane microdomains. *The Journal of Biological Chemistry*.

[70] Nishiga M., Horie T., Kuwabara Y. (2017). MicroRNA-33 controls adaptive fibrotic response in the remodeling heart by preserving lipid raft cholesterol. *Circulation Research*.

[71] Gurol T., Zhou W., Deng Q. (2016). MicroRNAs in neutrophils: potential next generation therapeutics for inflammatory ailments. *Immunological Reviews*.

